# Incidence of prevalent diseases in infants in Primary Health Care: a cohort study

**DOI:** 10.1590/1980-220X-REEUSP-2024-0358en

**Published:** 2025-05-30

**Authors:** Luana Bartsch, Veronica Souza Cavalheiro, Keli Verissimo Couto, Débora Cristina Limberger, Aline Cammarano Ribeiro, Darielli Resta Fontana, Leonardo Bigolin Jantsch

**Affiliations:** 1 Universidade Federal de Santa Maria, Palmeira das Missões, RS, Brazil.; 2 Universidade Federal de Santa Maria, Santa Maria, RS, Brazil.

**Keywords:** Child Care, Infant, Primary Health Care, Integrated Management of Childhood Illness

## Abstract

**Objective:**

To analyze the health problems prevalent in infants in the first year of life in a municipality in northern Rio Grande do Sul.

**Method:**

This is a retrospective, documentary cohort study, with a search in electronic medical records of Primary Health Care services for live births in 2022. These participants were followed through access to the electronic medical record report from January 29, 2022 to December 30, 2023, excluding the neonatal period. Data were analyzed using frequency comparison.

**Results:**

More than half of the children monitored in the cohort received at least one treatment for a prevalent childhood condition; approximately 43% received two treatments; and 30% received three treatments in the first year of life in Primary Care. Most of the problems are associated with respiratory conditions, begin after the 2nd month of life, and persist until the 10th month.

**Conclusion:**

The importance of continuous monitoring and appropriate preventive and therapeutic interventions during the first year of life and at critical moments is highlighted.

## INTRODUCTION

Primary Health Care (PHC), initially considered the gateway to the health system, has taken on a prominent role, more precisely in the Health Care Network (*RAS*), acting as a central point of communication, organization, and coordination of health care in Brazil. In this context, the priority strategy for expanding, strengthening, and qualifying PHC is the Family Health Strategy (FHS). This has achieved significant results in health, efficiency, and equity indicators^(1)^. However, the level of resolution of the PHC has to be known, as it influences the individuals’ health-disease process, allowing the identification of failures in care that can result in health complications and hospitalizations, when they are not resolved and embraced^(2)^.

In the context of child health, within PHC, it is recognized that the main health problems are related to the assessment of child development and growth, as well as to attention to problems prevalent in childhood^(3)^, situations that are also recognized within childcare routines or from spontaneous demands.

Caring for children in early childhood is important, as this is the stage in which the nervous system develops and skills are acquired, making it easier to develop future abilities. Furthermore, the relevance of the neonatal period (zero to 28 days) and the first thousand days, a phase of unique neurological, emotional, cognitive, and psychomotor development, is highlighted. Nevertheless, it is in this age group that the child may be susceptible to illnesses and injuries^(4)^.

Child health has been a priority on Brazil’s political agendas for decades, resulting in significant advances. An important milestone in this context was the introduction, in 1996, of the Comprehensive Management of Childhood Illness (*AIDPI*) strategy, which is used to monitor child health care in PHC. This strategy aims to encourage health promotion, disease prevention, early detection and effective treatment of the main problems that affect children’s health. The implementation of AIDPI has been associated with a significant reduction in infant mortality rates from these diseases, and has helped in the full development of children, supporting research to identify and treat childhood illnesses that converge, for the most part, with Hospitalizations for Primary Care-Sensitive Conditions (ICSAP)^(3)^.

In 2015, significant progress in child health was achieved with the publication of Ordinance No. 1.130, which established the National Policy for Comprehensive Child Health Care (PNAISC). PNAISC is structured into axes. Axis IV stands out: “Comprehensive care for children with prevalent childhood illnesses and chronic diseases”, in which childhood illnesses are discussed from a broader perspective, as well as chronic diseases, with the aim of developing strategies, early diagnosis and providing qualified care^(5,6)^.

According to the AIDPI strategy, a set of diseases with a higher incidence in childhood are considered prevalent diseases. The most common are: respiratory problems, such as coughing or difficulty breathing, which are associated with pneumonia, wheezing, acute or chronic ear infections and throat infections; febrile problems, including febrile illnesses and malaria in high-risk areas; diarrheal problems, such as dehydration, persistent diarrhea, and dysentery; and nutritional problems, related to malnutrition, anemia, and other growth problems^(7)^.

Among these conditions, the literature indicates that respiratory diseases are within the main causes of morbidity in children in Brazil, being highlighted as causes of hospital admissions due to conditions sensitive to PHC. This underscores the importance of a robust and effective PHC system for the prevention and management of these diseases, thus reducing the need for hospitalizations^(8)^. It should be emphasized that the literature presents the AIDPI strategy as a practice of the nursing professional during childcare and that there is still a prevalence of infant deaths due to preventable causes that could be prevented and treated with health actions and qualified conduct based on AIDPI^(9)^. However, to do so, the main health problems seen have to be identified, as well as their incidence and risk factors for the population under 1 year of age, the age period of greatest vulnerability in childhood^(10)^. To this end, the present study aims to analyze the health problems prevalent in infants in the first year of life in a municipality in northern Rio Grande do Sul.

## METHOD

### Design of Study

This is a study originating from a matrix research project entitled “Health Problems and follow-up of infants in early childhood: a cohort study”, which is an ambidirectional (retrospective and prospective) and documentary cohort study.

### Population, Local and Selection Criteria

The study local is the municipality of Palmeira das Missões, in the northwest region of the state of Rio Grande do Sul, with a population of 33,216 inhabitants^(11)^. The municipal health care network for the population is made up of 11 FHSs, which represent 100% coverage.

This is a population-based study including as participants the cohort of live births from the municipality of Palmeira das Missões, RS, who were born in the year 2022 (January 1 to December 31, 2022) and resided in the municipality at the time of data collection. According to the report made available by the Municipal Health Department, there were 383 births of residents of Palmeira das Missões in 2022 (N = 383).

Participants were recruited through the list of live births (containing the child’s full name, mother’s name, and date of birth). Those who, at the time of data collection, had a residential address outside the study local were excluded, totaling, at the end of data collection, 350 participants (N = 350).

### Data Collection

Data were collected through access to electronic medical records in the municipality’s health services, E-SUS (PHC medical records). For the purpose of this study, follow-up data and childhood injuries were collected at each access/record in the participants’ electronic medical records, from January 29, 2022 to December 30, 2023, considering that this was the period in which the children were between 28 and 365 days old, with the aim of excluding the neonatal period. The platform used as a tool for data collection was *Google Forms,* which electronically generates an Excel spreadsheet.

The data collection instrument was a semi-structured form constructed based on the Ministry of Health manuals^(12)^, AIDPI manual (version 2012 (neonatal) and 2017 version (2 months to 5 years)) and relevant literature in the area^(13)^. The variables used were: demographic (urban/rural housing) and maternal (maternal age) characterization; sex (male and female) and color/race (white/non-white) of the child; recommended number and frequency of follow-up consultations in childcare; care professional; and injuries in early childhood, with the outcome analyzed in this study (development of an injury prevalent in the first year of life), classified according to the International Classification of Primary Care (ICPC) or International Classification of Diseases (ICD) provided by the professional who provided the care. This ICD or ICPC was classified according to the conditions described in the AIDPI manual by the study researchers, under the supervision of the researcher in charge. As this is a documentary study, based on analysis of PHC care records, follow-up losses were not considered.

### Data Analysis

Data were analyzed, from the perspective of characterization, in a descriptive format (relative frequency (%) and absolute frequency (n)). To compare independent variables, such as maternal age, place of residence, color/race and frequency of follow-up consultations in childcare, with the outcomes analyzed in this study (development of a prevalent condition in the first year of life), frequency comparison and Relative Risk were used, depending on the characteristic of the variable, as well as mean comparison and T-test, defined after testing the normality of the data. To compare the age of the first injury with the type of injury, conduct and care professional, the Kruskal-Wallis test was used.

### Ethical Aspects

The project was approved by the Research Ethics Committee of the Universidade Federal de Santa Maria, *Campus* Palmeira das Missões, under Opinion No. 6.494.336 and Certificate of Presentation for Ethical Appreciation 74518823.9.00005346, in October 2023. The research complies with Resolution No. 466, of December 12, 2012, and the General Law on the Protection of Personal Data, Law No. 13.709/2018, especially what governs its article 7 (“IV - for carrying out studies by a research body, ensuring, whenever possible, the anonymization of personal data”). The Free and Informed Consent Form was used as the consent instrument.

## RESULTS

Of the 350 medical records analyzed, 52% are of female children, and the majority of race/color was white (96%), residing in the urban area of the municipality (94%). The mean maternal age was 28 years (SD ± 6.5 years). Regarding the characterization of care provided from 28 to 364 days of life in this follow-up cohort, Table 1 describes the number of care provided and the average age at care.

**Table 1:** Characterization of the number of visits in the first year of life and the classification of injuries according to comprehensive care for prevalent childhood diseases – Palmeira das Missões, RS, Brazil, 2024.

Number of appointments	n(%)	Average (months)	Minimum-maximum (months)
One consultation	192(54.9)	4.9(3.1)	1–11.7
Two consultations	150(42.9)	5.8(2.9)	1.1–11.9
Three consultations	105(30.0)	7.0(2.9)	1.4–11.76
Four consultations	66(18.9)	7.5(3.1)	1.11
Five consultations	45(12.9)	8.3(2.6)	0.9–11.37
Six consultations	20(5.7)	8.6(2.9)	2.9–11.9
Seven consultations	11(3.1)	8.3(3.0)	3.3–11.6
Eight consultations	8(2.3)	8.1(2.9)	3.7–11.7
Nine consultations	5(1.4)	8.7(1.7)	6.1–10.2
Ten or more consultations	17(4.8)	9.9(1.5)	8.3–11.9
**Injuries (Comprehensive Care for Prevalent Childhood Illnesses)**			
Respiratory dysfunction	283(52.4)		
Diarrhea	30(5.6)		
Fever	45(8.3)		
Ear problem	12(2,2)		
Sore throat	11(2.0)		
Malnutrition/anemia/growth problem	14(2.6)		
Allergies	35(6.5)		
Others	110 (20.4)		

More than half of the children monitored in the cohort received at least one treatment for a condition prevalent in childhood; approximately 43% received two treatments; and 30% received three treatments in the first year of life. Most of the injuries are associated with respiratory dysfunction. Regarding the type of injury and professional who provided the care, Table 2 stands out.

**Table 2:** Relationship between the condition in comprehensive care for prevalent childhood illnesses and the professional who provided the service in primary health care – Palmeira das Missões, RS, Brazil, 2024.

Condition	Nurse	FHS doctor	Pediatrician	Total	p value*
Respiratory dysfunction	54(19.1)	187(66.1)	42(14.8)	283(100.0)	<0.001
Diarrhea	5(16.7)	21(70.0)	4(13.3)	30(100.0)	
Fever	9(20.0)	28(62.2)	8(17.8)	45(100.0)	
Ear problem	1(8.3)	7(58.3)	4(33.3)	12(100.0)	
Sore throat	5(45.5)	6(54.5)	0(0.0)	11(100.0)	
Malnutrition/anemia/growth problem	3(21.4)	7(50.0)	4(28.6)	14(100.0)	
Allergies	12(34.3)	16(45.7)	7(20.0)	35(100.0)	
Others	45(40.09)	49(44.5)	16(14.5)	110(100.0)	
**Total**	**134(24.8)**	**321(59.4)**	**85(15.7)**	**540(100.0)**	

Legend: FHS – Family Health Strategy;

*Chi-square test.

In the analysis between the AIDPI condition and the professional who provided the care, it is highlighted that the FHS medical professional treated more problems due to respiratory dysfunction (66.1), diarrhea (70.0), fever (62.2) and ear problems (58.3). The nursing professional performed more care related to sore throat (45.5) and allergies (34.3). Meanwhile, the pediatrician attended to complaints of ear problems (33.3) and malnutrition/anemia/growth problems (28.6). In total, the FHS medical professional performed the largest number of consultations, with almost 60% of consultations, followed by the nurse, with approximately 25%, and the pediatrician, with 15.7%. Table 3 describes the risk factors identified for the development of at least one condition in the first year of PHC care.

**Table 3:** Risk factors for the development of health problems in the first year of life – Palmeira das Missões, RS, Brazil, 2024.

	Condition 1st year		RR(CI)	p value*
	Yes	No		
Neonatal injury*				<0.001
*Yes*	3(9,4)	29(90,6)	1	
*No*	189(59.4)	129(40.6)	14.163 (4.225–47.474)	
Sex				0.933
*Male*	92(55.1)	75(44.9)	1.018(0.668–1.552)	
*Female*	100(54.6)	83(45.4)	1	
Place of residence				
*Urban*	185(56.4)	143(43.6)	2.772(1.101–6.976)	0.025
*Rural*	7(31.8)	15(68.2)	1	
Race/color				0.706
*White*	183(55.1)	149(44.9)	1	
*Non-white*	7(50.0)	7(50.0)	0.814(0.279–2.373)	
Childcare in the 1st month*	53(39.6)	81(60.4)	1.446(0.934–2.238)	0.061
Childcare in the 2nd month*	75(65.2)	40(34.8)	1.891(1.192–2.999)	0.006
Childcare in the 4th month*	53(66.3)	27(33.8)	1.850(1.098–3.116)	0.020
Childcare in the 6th month*	55(65.5)	29(34.5)	1.7886(1.072–2.974)	0.025
Childcare in the 9th month*	40(72.7)	15(27.3)	2.509(1.328–4.738)	0.004
Childcare in the 12th month*	63(71.6)	25(28.4)	2.598(1.540–4.382)	<0.001

Legend: *Having developed a condition treated in PHC during the neonatal period;

**Having had an adequate number of consultations as per the guidelines of the Ministry of Health, Brazil;

***Chi-square test; CI – Confidence Interval; RR – Relative Risk.

Children who did not develop any health problems during the neonatal period have a 14 times greater risk of developing a health problem during the first year of life. There is also a significant difference in terms of place of residence, with a risk approximately 3 times greater for those living in urban areas, when compared to those living in rural areas. Regarding childcare monitoring, it is clear that children with a greater number of childcare consultations also presented an increased risk of receiving care for health problems. Table 4 shows the procedures adopted for each case of care in the first year of life and the professional who guided the procedure.

**Table 4:** Procedures adopted to deal with problems in the first year of life according to the professional who guided it – Palmeira das Missões, RS, Brazil, 2024.

Professional conduct		
Condition	Episode discharge (no referral)	Referred to childcare or specialized service
Respiratory dysfunction	16(65.2)	89 (34.8)
Diarrhea	21(70.0)	9(30.0)
Fever	34(77.3)	10(22.7)
Ear problem	7(58.3)	5(41.7)
Sore throat	8(72.7)	3(27.3)
Malnutrition/anemia/growth problem	6(50.0)	6(50.0)
Allergies	17(58.6)	12(41.4)
Others	46(44.2)	58(55.8)
**Professional who performed the service**		
Family Health Strategy Physician	259(78.5)	71(21.5)
Nurse	54(39.7)	82(60.3)
Pediatrician	31(34.8)	58(65.2)

**Figure 1:**
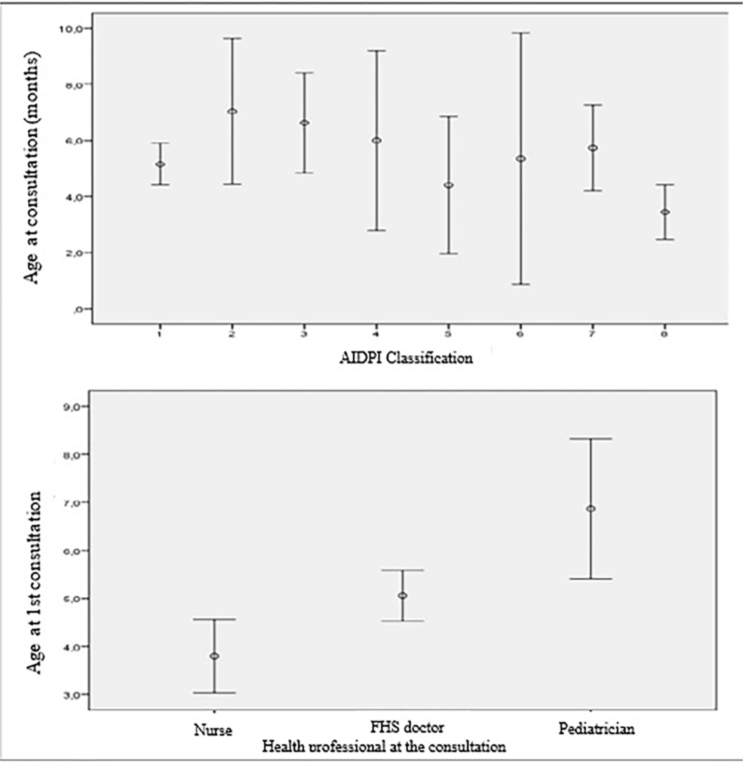
Relationship between age at consultation, condition and professional who provided care at the first consultation. Palmeira das Missões, Rio Grande do Sul, Brazil, 2024.

The procedures for care vary according to the classification of the condition by AIDPI. The vast majority of procedures were discharge from the episode (without referral) for respiratory dysfunction (65.2), diarrhea (70.0), fever (77.3), ear problem (58.3) and sore throat (72.7). Malnutrition/anemia/growth problems accounted for 50% of cases of discharge and referral for follow-up in childcare, with the condition “others” (55.8) being the most frequently referred for follow-up in childcare or specialized services. Figure 1 shows the age in the consultation related to the condition in the first year of life and to the professional who provided the care.

Acronym: 1- respiratory; 2 – diarrhea; 3 – fever; 4 – ear problem; 5 – sore throat; 6 – malnutrition/anemia/growth problem; 7 – allergies; 8 – others.

Health problems in children show different prevalence patterns throughout the first months of life. It is observed that respiratory dysfunction is concentrated, on average, in the 5th month of life. Malnutrition/anemia/growth problems start at the 1st month and extend until the 10th month, with a peak prevalence in the 6th month. Diarrhea, fever and ear problems are more common from the 6th to the 8th month of life, and sore throat is more prevalent in the 4th month. Ages at first consultation vary significantly (p < 0.001), depending on the professional. Consultations with pediatricians tend to occur later (on average) compared to consultations with nurses and ESF doctors.

## DISCUSSION

More than half of the infants had at least one consultation at the FHS for some health problem in the first year of life, the majority of which were associated with respiratory problems, with a predominance of consultations carried out by the FHS doctor. Factors such as having a condition in 1st month of life and living in rural areas were considered protective. Being monitored by a childcare provider is essential for carrying out consultations regarding childhood problems in the first year of life. Nurses are the professionals who care for younger infants, when compared to the demands of FHS doctors and pediatricians. Regarding the referrals made, the results converge with the low adherence to childcare follow-up by FHS doctors, reflecting the quality of undergraduate training, the current model, which may not cover all the essential skills for adequate child health care in PHC, in addition to the absence or ineffectiveness of continuing education programs during professional practice. Furthermore, the practice of referrals for follow-up in preventive medicine and childcare by family doctors is still in its infancy^(14)^.

Childhood is a period in which many problems can arise. More than half of the infants had at least one visit for a medical condition in the first year of life, and the majority were associated with respiratory conditions, according to the findings of this study. In the literature, acute respiratory infections represent one of the main causes of morbidity and mortality in childhood, and the South region has the highest rates of respiratory diseases, due to the seasonality of the climate^(15)^, corroborating the study, which highlights respiratory diseases as the main causes of morbidity in children in Brazil^(8)^.

Children under 1 year old have the highest number of hospitalizations from respiratory diseases, due to the immaturity of the immune system, characteristics of the respiratory system itself in the first 12 months of life, interruption of breastfeeding, and beginning of activities in daycare centers^(13,14)^. According to this study, approximately 81.5% of infants had some health problem in the first year of life, and 93.3% in the second year of life, with the most frequent problems being related to the digestive and respiratory systems. Furthermore, 24.7% of infants aged 2 years had already been hospitalized at least once, and children who were not exclusively breastfed until 6 months or who did not receive breast milk until the first year of life had a higher predominance of prevalent diseases^(16)^.

Meanwhile, international literature in the United Kingdom highlights that the majority of care for children under 5 years of age in PHC is for acute and self-limiting viral conditions, that is, they are resolved without medical intervention and could be managed by health professionals, and the most common reason for hospitalizations was also due to upper respiratory tract infections (14%) and cough (7%)^(17)^.

Globally, there are an estimated 33 million episodes of acute lower respiratory tract infection associated with respiratory syncytial virus (RSV) in children aged 0 to 60 months. Infants are affected in all regions, but the incidence of acute RSV-associated lower respiratory tract infection peaked in low- and lower-middle-income countries in infants under 3 months and in upper-middle- and high-income countries in infants aged 3 to 6 months. An average of five months is observed, a period with the highest incidence of respiratory problems, corroborating the beginning of many of them in daycare centers/schools due to the return of mothers to work^(18)^. A study of 51,292 children in Spain, under 5 years of age, also showed that, in the first year of life, there was the highest number of acute respiratory infections and RSV (134.4 per 1,000 children)^(19)^.

Conversely, it is important to highlight the effectiveness of PHC, as it has a great impact on prevention and early identification of the outcome of these illnesses, especially respiratory illnesses in children^(15)^. It is worth noting that diseases prevalent in childhood can be identified early through childcare, which should carry out early identification of signs of the main problems and recommend the most effective actions, forming a bond with the family and having the opportunity, as a nurse, to support and guide the family towards early recognition of problems^(16)^.

Meanwhile, the literature points to the challenges in implementing AIDPI, which despite having been introduced a long time ago, is still not widely adopted in the management of prevalent childhood diseases within the PHC framework. The application of this strategy faces obstacles globally, as indicated in international studies, due to its underutilization in the diagnosis and treatment process, and in motivational factors of a personal, management and political-economic nature. These obstacles result in less than desired care for children under 5 years of age, impacting morbidity and mortality rates due to preventable causes, which could be treated effectively in PHC^(20,21)^.

The organization of PHC around FHSs expanded access and achieved good results given the complexity of the population’s health-disease process. However, PHC in Brazil faces challenges, such as the maintenance of characteristics of the traditional model, characterized by emergency care as a reference, and weaknesses in care coordination, family-centered care and longitudinality. There are many restrictions, and most of them refer to the devaluation of the Brazilian Public Health System, the lack of motivation in professional training, the lack of financial resources, among others^(22)^.

Countries that successfully implement the AIDPI strategy report that evidence-based treatment improves the quality of care, as well as the identification of signs and symptoms for professional decision-making. AIDPI has been shown to be effective in improving the quality of care, increasing health cost savings and potentially reducing child mortality in developing countries. Structured care, through comprehensive and consistent integrated management of childhood (AIDPI), has been used to promote accurate assessment and classification of childhood illnesses, in addition to ensuring appropriate combined treatment, providing caregivers with advice and resoluteness, especially in referrals, and directly contributing to the reduction of infant mortality and morbidity^(23)^.

The rural environment was a protective factor against the development of injuries. It is internationally evident that, in territories with rural areas where care is provided by nurses, AIDIP is used and has been praised for systematically identifying danger signs and referring these children when necessary, proving to be effective^(20)^. In Brazil and in some countries, the findings differ, as residents of rural areas have poor access to care, and have worse health conditions when compared to the urban population. The difficulties highlighted include transportation for locomotion and communication. First, local options are exhausted, and then health care is sought outside the community. Conversely, it is observed that, in some communities, the rural population has access to nearby health services, but chooses to travel to larger centers to seek treatment^(24)^. This shift may also be linked to the quality of care, especially medical care provided to the population in rural settings. An international study indicates that, in these services, inadequate diagnoses, irrational use of antibiotics and other medications and polypharmacy were considered standard for medical practice in PHC, which deviates from the promotion and prevention model and conduct towards the curative care model^(25)^.

It was evident that having an injury in the neonatal period can be considered a protective factor as these children can be recognized/seen by the team and, thus, reduce the risk of worsening or development of injuries throughout the first year of life, in addition to highlighting the nurse as a care professional in the first months. In this study, it is observed that 57.1% of caregivers consistently use the same health service to care for their children, and show a significant preference for the nurse as the professional who knows them best^(26)^. Nurses are seen as competent and accessible professionals, capable of providing appropriate guidance and establishing a relationship of trust and bond with caregivers^(8)^.

For the present study, it was observed that children with regular childcare monitoring had more recorded injuries in the first year of life. This monitoring is essential for the early identification of health problems and timely intervention, since childcare strengthens the recognition of these grievances. In the literature, childcare in PHC makes it clear that FHS is identified as a reference service by most caregivers, but there is still a high rate of other services in the care network seen as a reference. When new health problems arise, there is a tendency to seek another service for resolution, which may indicate issues related to the perception of the quality of specialized care in the usual service^(26)^. It is also concerning that most of the medical records were related to childcare, which shows that the municipality’s PHC services were not sought due to complaints of complications/health problems^(16)^. Behaviors like these highlight the importance of strengthening PHC and ensuring comprehensive care to avoid fragmentation of services^(26)^.

This corroborates international studies, which show that many parents ignore primary care and prefer to take their children to specialists (secondary or tertiary level) and use invasive methods, such as hospital-centered and medicalized treatment, with, in many cases, Primary Health Units being used for immunization, monitoring of healthy people and preventive measures^(20)^.

The limitations of the study are linked to the finding of incomplete medical records and/or lack of information in the reports extracted from the medical records and/or their non-existence. Another limiting factor is the identification of only those conditions that were embraced, attended to and registered in the PHC, through the FHS, excluding those that were made invisible by the FHS service or that were received at other points in the RAS, which makes it impossible to generalize to all children in the geographic setting of the study.

## CONCLUSION

The respiratory problems were the most prevalent in the first year of life and classified as sensitive to PHC. Most health problems begin after the 2nd month of life and persist until the 10th month. This pattern indicates the importance of continued surveillance and appropriate preventive and therapeutic interventions during the first year of life to address these health problems at critical times. There are protective factors to that population, such as living in rural areas of the municipality as well as having a problem in the first days of life, which allows children’s visibility and follow-up in the childcare program in the FHS.

Preventive interventions, such as regular childcare monitoring, can facilitate the identification of the condition, appropriate treatment, and continuous monitoring of children who present with conditions in the 1st month of life, being essential to improve children’s health. It is clear that health teams, especially nurses, need to strengthen training to improve care practices for newborns and infants, especially regarding AIDPI actions, since most care was provided by the FHS doctor. The health team, especially FHS doctors, needs to recognize the importance of, upon completing the care for the condition, transferring this child to childcare routines and systematic care, recognizing possible risks to the child’s growth and development.

It is necessary to organize public health services, with the development of resolutive strategies, based on the diagnosis established by this study, and that actions to prevent and strengthen PHC can be recognized early and be problem-solving, preventing complications, ICSAP and the weak implementation of AIDPI.

## Data Availability

Data is available upon request/contact with the corresponding author.
