# Supplementary material for: Incidence of prevalent diseases in infants in Primary Health Care: a cohort study
Source: Rev Esc Enferm USP. 2025 May 30;59:e20240358. doi: 10.1590/1980-220X-REEUSP-2024-0358en (PMC12129401; doi:10.1590/1980-220X-REEUSP-2024-0358en)
Supplement: Translation

# Translation: Incidência de agravos prevalentes em lactentes na Atenção Primária à Saúde: estudo de coorte

**DOI:** 10.1590/1980-220X-REEUSP-2024-0358pt

**Authors:** Luana Bartsch, Veronica Souza Cavalheiro, Keli Verissimo Couto, Débora Cristina Limberger, Aline Cammarano Ribeiro, Darielli Resta Fontana, Leonardo Bigolin Jantsch

**Affiliations:** 1 Universidade Federal de Santa Maria, Palmeira das Missões, RS, Brasil.; 2 Universidade Federal de Santa Maria, Santa Maria, RS, Brasil.

**Keywords:** Cuidado da Criança, Lactente, Atenção Primária à Saúde, Atenção Integrada às Doenças Prevalentes na Infância

## Abstract

**Objetivo:**

Analisar os agravos de saúde prevalentes em lactentes no primeiro ano de vida em um município do norte gaúcho.

**Método:**

Trata-se de estudo de coorte de caráter retrospectivo, documental, com busca em prontuários eletrônicos dos serviços de saúde da Atenção Primária à Saúde dos nascidos vivos no ano de 2022. Esses participantes foram acompanhados por meio de acesso ao relatório dos prontuários eletrônicos no período de 29 de janeiro de 2022 a 30 de dezembro de 2023, excluindo o período neonatal. Os dados foram analisados sob comparação de frequência.

**Resultados:**

Mais da metade das crianças acompanhadas na coorte obteve no mínimo um atendimento por agravo prevalente na infância; aproximadamente 43% tiveram dois atendimentos; e 30% tiveram três atendimentos no primeiro ano de vida na Atenção Primária. Em sua maioria, os agravos estão associados a quadros respiratórios, iniciam após o 2º mês de vida e persistem até o 10º mês.

**Conclusão:**

Indica-se a importância de uma vigilância contínua e de intervenções preventivas e terapêuticas adequadas durante o primeiro ano de vida e em momentos críticos.

## INTRODUÇÃO

A Atenção Primária à Saúde (APS), considerada inicialmente como a porta de entrada do sistema de saúde, assume lugar de evidência, mais precisamente na Rede de Atenção à Saúde (RAS), atuando como ponto central de comunicação, organização e coordenação do cuidado em saúde no Brasil. Nesse contexto, a estratégia prioritária de expansão, fortalecimento e qualificação da APS é a Estratégia Saúde da Família (ESF). Esta tem alcançado resultados significativos nos indicadores de saúde, eficiência e equidade^(1)^. Mas é necessário conhecer o nível de resolutividade da APS, pois influencia o processo de saúde-doença dos indivíduos, permitindo identificar as falhas na assistência que podem resultar em complicações à saúde e hospitalizações, quando não são resolvidas e acolhidas^(2)^.

No contexto da saúde infantil, dentro da APS, reconhece-se que os principais agravos de saúde estão relacionados à avaliação do desenvolvimento e crescimento infantil, bem como atenção aos agravos prevalentes na infância^(3)^, situações essas reconhecidas também dentro das rotinas da puericultura ou demandas espontâneas.

O cuidado com a criança na primeira infância é importante, visto que nessa fase ocorrem o desenvolvimento do sistema nervoso e a aquisição de capacidades, com maior facilidade para o desenvolvimento de habilidades futuras. Além disso, destaca-se a relevância do período neonatal (zero a 28 dias) e dos primeiros mil dias, fase de desenvolvimento único neurológico, emocional, cognitivo e psicomotor. Entretanto, é nessa faixa etária que a criança pode estar suscetível a doenças e agravos^(4)^.

A saúde da criança tem sido uma prioridade nas agendas políticas do Brasil há décadas, resultando em avanços significativos. Um marco importante nesse contexto foi a introdução, em 1996, da estratégia de Atenção Integral às Doenças Prevalentes da Infância (AIDPI), que é utilizada para o seguimento do cuidado da saúde infantil na APS. Essa estratégia visa fomentar ações de promoção da saúde, prevenção de doenças, detecção precoce e tratamento efetivo dos principais agravos que afetam a saúde infantil. A implementação da AIDPI tem sido associada a uma redução significativa nas taxas de mortalidade infantil por essas doenças, e vem auxiliando no pleno desenvolvimento infantil, sendo um suporte de pesquisa para identificar e tratar os agravos infantis que convergem, em sua maioria, com as Internações por Condições Sensíveis à Atenção Primária (ICSAP)^(3)^.

No ano de 2015, um avanço significativo na saúde da criança foi alcançado com a publicação da Portaria nº 1.130, que estabeleceu a Política Nacional de Atenção Integral à Saúde da Criança (PNAISC). A PNAISC está estruturada em eixos. Destaca-se o eixo IV “Atenção integral a crianças com agravos prevalentes na infância e com doenças crônicas”, em que são discutidos os agravos da infância com olhar ampliado, bem como as doenças crônicas, com o intuito de desenvolver estratégias, diagnóstico precoce e proporcionar cuidado qualificado^(5,6)^.

De acordo com a estratégia AIDPI, são considerados agravos prevalentes um conjunto de doenças com maior incidência na infância. Os mais comuns são: agravos respiratórios, como tosse ou dificuldade para respirar, que estão associados à pneumonia, sibilância, infecção aguda ou crônica do ouvido e infecção de garganta; agravos febris, incluindo doenças febris e malária em áreas de risco; agravos diarreicos, como desidratação, diarreia persistente e disenteria; e agravos nutricionais, relacionados à desnutrição, anemia e outros problemas de crescimento^(7)^.

Entre esses agravos, a literatura indica que as doenças respiratórias estão entre as principais causas de morbidade em crianças no Brasil, sendo destacadas como causas de internações hospitalares por condições sensíveis à APS. Isso ressalta a importância de um sistema de APS robusto e eficaz para a prevenção e manejo dessas doenças, reduzindo, assim, a necessidade de hospitalizações^(8)^. Vale destacar que a literatura traz a estratégia AIDPI como prática do profissional enfermeiro durante a puericultura e que ainda há prevalência de óbitos infantis por causas evitáveis que poderiam ser prevenidas e tratadas com ações de saúde e conduta qualificada baseada na AIDPI^(9)^. Mas, para isso, é necessário identificar os principais agravos acolhidos, sua incidência e fatores de risco para a população de menores de 1 ano, período etário de maior vulnerabilidade na infância^(10)^. Para isso, o presente estudo tem como objetivo analisar os agravos de saúde prevalentes em lactentes no primeiro ano de vida em um município do norte gaúcho.

## MÉTODO

### Tipo de Estudo

Trata-se de estudo originado de projeto de pesquisa matricial intitulado “Agravos de Saúde e seguimento de lactentes na primeira infância: um estudo de coorte”, sendo este um estudo de coorte ambidestra (retrospectiva e prospectiva) e documental.

### Local, População e Critérios de Seleção

O local do estudo refere-se ao município de Palmeira das Missões, localizado na região noroeste do Rio Grande do Sul, com população de 33.216 habitantes^(11)^. A rede municipal de atenção à saúde da população é constituída por 11 ESFs, o que representa uma cobertura de 100%.

Trata-se de estudo de caráter populacional que incluiu como participantes a coorte de nascidos vivos, do município de Palmeira das Missões, RS, que nasceram no ano de 2022 (01 de janeiro a 31 de dezembro de 2022) e residiam no município no momento da coleta dos dados. Conforme o relatório disponibilizado pela Secretaria Municipal de Saúde, foram 383 nascimentos de residentes em Palmeira das Missões no ano de 2022 (N = 383).

Os participantes foram recrutados por meio de acesso da lista de nascidos vivos (contendo nome completo da criança, nome da mãe e data de nascimento). Foram excluídos aqueles que, no momento da coleta dos dados, possuíam endereço de residência fora do município, cenário do estudo, totalizando, ao final da coleta de dados, 350 participantes (N = 350).

### Coleta de Dados

Os dados foram coletados por meio de acesso aos prontuários eletrônicos nos serviços de saúde do município, E-SUS (prontuário da APS). Para o recorte deste estudo, os dados de seguimento e agravos na infância foram coletados a cada acesso/registro nos prontuários eletrônicos dos participantes, no período de 29 de janeiro de 2022 a 30 de dezembro de 2023, tendo em vista que esse foi o período em que as crianças tiveram entre 28 e 365 dias de vida, com o intuito de excluir o período neonatal. Utilizou-se como ferramenta para a coleta dos dados a plataforma *Google Forms,* que gera eletronicamente uma planilha *Excel*.

O instrumento para coleta de dados foi um formulário semiestruturado construído com base nos manuais do Ministério da Saúde^(12)^, manual da AIDPI (versão 2012 (neonatal) e versão 2017 (2 meses a 5 anos)) e literatura pertinente na área^(13)^. As variáveis utilizadas foram: caracterização demográfica (moradia urbana/rural) e materna (idade materna); sexo (masculino e feminino) e cor/raça (brancos/não brancos) da criança; número e frequência recomendados de consultas de seguimento na puericultura; profissional de atendimento; e agravos na primeira infância, com o desfecho analisado neste estudo (desenvolvimento de agravo prevalente no primeiro ano de vida), classificados conforme Classificação Internacional de Atenção Primária (CIAP) ou Classificação Internacional de Doenças (CID) disponibilizado pelo profissional que realizou o atendimento. Esse CID ou CIAP foi classificado conforme os agravos descritos no manual da AIDPI pelos pesquisadores do estudo, sob supervisão do pesquisador responsável. Por tratar-se de estudo documental, sob análise dos registros de atendimento na APS, perdas de seguimento não foram consideradas.

### Análise dos Dados

Os dados foram analisados, sob a perspectiva de caracterização, em formato descritivo (frequência relativa (%) e frequência absoluta(n)). Para comparação das variáveis independentes, como idade materna, local da residência, cor/raça e frequência das consultas de seguimento em puericultura, com os desfechos analisados neste estudo (desenvolvimento de agravo prevalente no primeiro ano de vida), foram utilizados comparação de frequência e Risco Relativo, dependendo da característica da variável, assim como comparação de média e teste T, definido após teste de normalidade dos dados. Para comparação da idade do primeiro agravo com o tipo de agravo, condutas e profissional de atendimento, foi utilizado o teste de Kruskal-Wallis.

### Aspectos Éticos

O projeto foi aprovado pelo Comitê de Ética em Pesquisa da Universidade Federal de Santa Maria, *Campus* Palmeira das Missões, sob Parecer nº 6.494.336 e Certificado de Apresentação para Apreciação Ética 74518823.9.00005346, em outubro de 2023. A pesquisa está em conformidade com a Resolução nº 466, de 12 de dezembro de 2012, e a Lei Geral de Proteção de Dados Pessoais, Lei nº 13.709/2018, especialmente o que rege seu artigo 7 (“IV - para a realização de estudos por órgão de pesquisa, garantida, sempre que possível, a anonimização dos dados pessoais”). Foi utilizado como instrumento de consentimento o Termo de Consentimento Livre e Esclarecido.

## RESULTADOS

Dos 350 prontuários analisados, 52% são de crianças do sexo feminino, e a raça/cor majoritariamente foi a branca (96%), residindo na área urbana do município (94%). A média da idade materna foi de 28 anos (DP ± 6,5 anos). Quanto à caracterização dos atendimentos dos 28 aos 364 dias de vida desta coorte de acompanhamento, a Tabela 1 descreve o número de atendimento e a média de idade nos atendimentos.

**Tabela 1:** Caracterização do número de atendimentos no primeiro ano de vida e a classificação dos agravos de acordo com atenção integral às doenças prevalentes da infância – Palmeira das Missões, RS, Brasil, 2024.

Número de atendimentos	n(%)	Média (meses)	Mínimo-Máximo (meses)
Um atendimento	192(54,9)	4,9(3,1)	1–11,7
Dois atendimentos	150(42,9)	5,8(2,9)	1,1–11,9
Três atendimentos	105(30,0)	7,0(2,9)	1,4–11,76
Quatro atendimentos	66(18,9)	7,5(3,1)	1,11
Cinco atendimentos	45(12,9)	8,3(2,6)	0,9–11,37
Seis atendimentos	20(5,7)	8,6(2,9)	2,9–11,9
Sete atendimentos	11(3,1)	8,3(3,0)	3,3–11,6
Oito atendimentos	8(2,3)	8,1(2,9)	3,7–11,7
Nove atendimentos	5(1,4)	8,7(1,7)	6,1–10,2
Dez ou mais atendimentos	17(4,8)	9,9(1,5)	8,3–11,9
**Agravos (Atenção Integral** às **Doenças Prevalentes da Infância)**			
Disfunção respiratória	283(52,4)		
Diarreia	30(5,6)		
Febre	45(8,3)		
Problema no ouvido	12(2,2)		
Dor de garganta	11(2,0)		
Desnutrição/anemia/problema de crescimento	14(2,6)		
Alergias	35 (6,5)		
Outros	110(20,4)		

Mais da metade das crianças acompanhadas na coorte obteve no mínimo um atendimento por agravo prevalente na infância; cerca de 43% tiveram dois atendimentos; e 30% tiveram três atendimentos no primeiro ano de vida. Em sua maioria, os agravos estão associados à disfunção respiratória. Quanto ao tipo de agravo e profissional que realizou o atendimento, destaca-se a Tabela 2.

**Tabela 2:** Relação entre o agravo na atenção integral às doenças prevalentes da infância e o profissional que realizou o atendimento na atenção primária à saúde – Palmeira das Missões, RS, Brasil, 2024.

Agravo	Enfermeiro	Médico da ESF	Pediatra	Total	Valor de p*
Disfunção respiratória	54(19,1)	187(66,1)	42(14,8)	283(100,0)	<0,001
Diarreia	5(16,7)	21(70,0)	4(13,3)	30(100,0)	
Febre	9(20,0)	28(62,2)	8(17,8)	45(100,0)	
Problema no ouvido	1(8,3)	7(58,3)	4(33,3)	12(100,0)	
Dor de garganta	5(45,5)	6(54,5)	0(0,0)	11(100,0)	
Desnutrição/anemia/problema de crescimento	3(21,4)	7(50,0)	4(28,6)	14(100,0)	
Alergias	12(34,3)	16(45,7)	7(20,0)	35(100,0)	
Outros	45(40,09)	49(44,5)	16(14,5)	110(100,0)	
**Total**	**134(24,8)**	**321(59,4)**	**85(15,7)**	**540(100,0)**	

Legenda: ESF – Estratégia Saúde da Família;

*Teste qui-quadrado.

Na análise entre o agravo AIDPI e o profissional que realizou o atendimento, destaca-se que o profissional médico da ESF atendeu mais agravos por disfunção respiratória (66,1), diarreia (70,0), febre (62,2) e problema no ouvido (58,3). O profissional enfermeiro realizou mais atendimentos relacionados à dor de garganta (45,5) e alergias (34,3). Enquanto isso, o profissional pediatra atendeu os agravos de problema no ouvido (33,3) e desnutrição/anemia/problema de crescimento (28,6). Na totalidade, o profissional médico da ESF desempenhou o maior número de atendimentos, com quase 60% dos atendimentos, seguido do enfermeiro, com aproximadamente 25%, e do pediatra, com 15,7%. A Tabela 3 descreve os fatores de risco identificados para o desenvolvimento de, no mínimo, um agravo no primeiro ano de vida de atendimento na APS.

**Tabela 3:** Fatores de risco para o desenvolvimento de agravos no primeiro ano de vida – Palmeira das Missões, RS, Brasil, 2024.

	Agravo 1º ano		RR(IC)	Valor de p***
	Sim	Não		
Agravo neonatal*				<0,001
*Sim*	3(9,4)	29(90,6)	1	
*Não*	189(59,4)	129(40,6)	14,163 (4,225–47,474)	
Sexo				0,933
*Masculino*	92(55,1)	75(44,9)	1,018(0,668–1,552)	
*Feminino*	100(54,6)	83(45,4)	1	
Local de residência				
*Urbano*	185(56,4)	143(43,6)	2,772(1,101–6,976)	0,025
*Rural*	7(31,8)	15(68,2)	1	
Raça/cor				0,706
*Branca*	183(55,1)	149(44,9)	1	
*Não branca*	7(50,0)	7(50,0)	0,814(0,279–2,373)	
Puericultura no 1º mês*	53(39,6)	81(60,4)	1,446(0,934–2,238)	0,061
Puericultura no 2º mês*	75(65,2)	40(34,8)	1,891(1,192–2,999)	0,006
Puericultura no 4º mês*	53(66,3)	27(33,8)	1,850(1,098–3,116)	0,020
Puericultura no 6º mês*	55(65,5)	29(34,5)	1,7886(1,072–2,974)	0,025
Puericultura no 9º mês*	40(72,7)	15(27,3)	2,509(1,328–4,738)	0,004
Puericultura no 12º mês*	63(71,6)	25(28,4)	2,598(1,540–4,382)	<0,001

Legenda: *Ter desenvolvido agravo atendido na APS no período neonatal;

**Estar com número de consultas adequadas conforme orientação Ministério da Saúde, Brasil;

***Teste qui-quadrado; IC – Intervalo de Confiança; RR – Risco Relativo.

Crianças que não desenvolveram agravos no período neonatal têm 14 vezes mais risco de ter agravo ao longo do primeiro ano de vida. Há também uma diferença significativa no que tange ao local de residência, com um risco aproximadamente 3 vezes maior para quem vive em áreas urbanas, quando comparado com quem vive em áreas rurais. No que se refere ao acompanhamento na puericultura, fica evidente que as crianças com maior número de consultas de puericultura também apresentaram risco aumentado para o atendimento de agravos. A Tabela 4 mostra as condutas adotadas para cada atendimento de agravo no primeiro ano de vida e o profissional que orientou a conduta.

**Tabela 4:** Condutas adotadas para o atendimento de agravos no primeiro ano de vida de acordo com o profissional que a orientou – Palmeira das Missões, RS, Brasil, 2024.

	Conduta do profissional	
Agravo	Alta do episódio (sem encaminhamento)	Encaminhado para puericultura ou serviço especializado
Disfunção respiratória	167(65,2)	89(34,8)
Diarreia	21(70,0)	9(30,0)
Febre	34(77,3)	10(22,7)
Problema no ouvido	7(58,3)	5(41,7)
Dor de garganta	8(72,7)	3(27,3)
Desnutrição/anemia/problema de crescimento	6(50,0)	6(50,0)
Alergias	17(58,6)	12(41,4)
Outros	46(44,2)	58(55,8)
**Profissional que realizou o atendimento**		
Médico da Estratégia Saúde da Família	259(78,5)	71(21,5)
Enfermeiro	54(39,7)	82(60,3)
Pediatra	31(34,8)	58(65,2)

As condutas dos atendimentos variam de acordo com a classificação do agravo pela AIDPI. A grande maioria das condutas foi de alta do episódio (sem encaminhamento) para disfunção respiratória (65,2), diarreia (70,0), febre (77,3), problema no ouvido (58,3) e dor de garganta (72,7). Desnutrição/anemia/problema de crescimento obtiveram percentual de 50% para alta do episódio e encaminhamento para seguimento na puericultura, sendo o agravo “outros” (55,8) o mais encaminhado para seguimento na puericultura ou serviço especializado. A Figura 1 mostra a idade na consulta relacionada ao agravo no primeiro ano de vida e ao profissional que realizou o atendimento.

**Figura 1:** Relação entre a idade na consulta, agravo e profissional que realizou o atendimento na primeira consulta. Palmeira das Missões, Rio Grande do Sul, Brasil, 2024.

Sigla: 1- respiratório; 2 – diarreia; 3 – febre; 4 – problema no ouvido; 5 – dor de garganta; 6 – desnutrição/anemia/problema de crescimento; 7 – alergias; 8 – outros.

Os agravos de saúde em crianças apresentam diferentes padrões de prevalência ao longo dos primeiros meses de vida. Observa-se que a disfunção respiratória se concentra, em média, no 5º mês de vida. Desnutrição/anemia/problema de crescimento iniciam no 1^o^ mês e estendem-se até o 10º mês, com um pico de prevalência no 6º mês. Diarreia, febre e problema no ouvido são mais comuns do 6º ao 8º mês de vida, e dor de garganta apresenta maior prevalência no 4º mês. As idades na primeira consulta variam significativamente (p < 0,001), dependendo do profissional. Consultas com pediatras tendem a ocorrer mais tarde (em média) em comparação com as consultas de enfermeiros e médicos da ESF.

## DISCUSSÃO

Mais da metade dos lactentes teve pelo menos uma consulta na ESF por algum problema de saúde no primeiro ano de vida, sendo a maioria associada a problemas respiratórios, com predomínio de consultas realizadas pelo médico da ESF. Fatores como ter agravo no 1^o^ mês de vida e viver no meio rural foram considerados protetores. Estar em acompanhamento na puericultura é um determinante para realização de consultas diante dos agravos infantis no primeiro ano de vida. Os enfermeiros são os profissionais que acolhem lactentes com menor idade, quando comparado às demandas de médicos da ESF e pediatras. Quanto aos encaminhamentos realizados, os resultados convergem com a baixa adesão ao seguimento da puericultura pelos médicos da ESF, reflexo da qualidade de formação na graduação, do modelo atual, que pode não contemplar todas as competências essenciais para a adequada assistência à saúde da criança na APS, além da ausência ou ineficácia de programas de educação permanente durante o exercício profissional. Ademais, a prática de encaminhamento para seguimento em medicina preventiva e puericultura por parte de médicos da família ainda se mostra incipiente^(14)^.

A infância é um período em que muitos agravos podem surgir. Mais da metade dos lactentes teve, no mínimo, um atendimento por agravo no primeiro ano de vida, e ainda a maioria estava associada a quadros respiratórios, de acordo com os achados deste estudo. Na literatura, as infecções respiratórias agudas representam uma das principais causas de morbimortalidade na infância, e a região Sul possui as maiores taxas de doenças respiratórias, devido à sazonalidade do clima^(15)^, corroborando o estudo, que destaca as doenças respiratórias como as principais causas de morbidade em crianças no Brasil^(8)^.

As crianças menores de 1 ano apresentam o maior número de internações por doenças respiratórias, em função da imaturidade do sistema imunológico, características do próprio aparelho respiratório nos primeiros 12 meses de vida, interrupção do aleitamento materno e início das atividades em creches^(13,14)^. De acordo com este estudo, aproximadamente 81,5% dos lactentes apresentaram algum problema de saúde no primeiro ano de vida, e 93,3%, no segundo ano de vida, sendo os agravos mais frequentes relacionados aos sistemas digestivo e respiratório. Além disso, 24,7% dos lactentes com 2 anos já haviam sido hospitalizados ao menos uma vez, e ainda as crianças não amamentadas exclusivamente até os 6 meses ou que não receberam leite materno até o primeiro ano de vida apresentaram maior predomínio de agravos prevalentes^(16)^.

Enquanto isso, a literatura internacional, no Reino Unido, destaca que a maioria dos atendimentos em menores de 5 anos na APS é por condições virais agudas e autolimitadas, ou seja, são resolvidas sem intervenção médica e poderiam ser gerenciadas por profissionais de saúde, e o motivo de internações mais corriqueiras também foi devido às infecções do trato respiratório superior (14%) e tosse (7%)^(17)^.

Globalmente, estimam-se 33 milhões de episódios de infecção aguda das vias aéreas inferiores associadas ao vírus sincicial respiratório (VSR) em crianças de 0 a 60 meses. Os lactentes são afetados em todas as regiões, mas a incidência aguda de infecção das vias aéreas inferiores associada ao VSR teve pico em países de baixa e média-baixa renda nos menores de 3 meses e países de renda média-alta e alta de 3 a 6 meses. Observa-se uma média de cinco meses, período com maior incidência de agravos respiratórios, corroborando o início de muitos em creches/escolas devido ao retorno das mães ao trabalho^(18)^. Estudo com 51.292 crianças na Espanha, menores de 5 anos, também evidenciou que, no primeiro ano de vida, houve o maior número de infecções respiratórias agudas e VSR (134,4 por 1.000 crianças)^(19)^.

Em contrapartida, é relevante destacar a efetividade da APS, pois ela tem grande impacto para prevenção e identificação precoce no desfecho desses adoecimentos, principalmente da ordem respiratória em crianças^(15)^. Destaca-se que as doenças prevalentes na infância podem ser identificadas precocemente por meio da puericultura, a qual deve realizar a identificação precoce de sinais dos principais agravos e recomendar as ações mais efetivas, formando o vínculo com a família e tendo a oportunidade, enquanto enfermeiro, de apoiar e orientar a família para o reconhecimento precoce dos agravos^(16)^.

Enquanto isso, a literatura aponta para os desafios na implementação da AIDPI, que apesar de ter sido introduzida há muito tempo, ainda não é amplamente adotada no manejo das doenças prevalentes na infância no âmbito da APS. A aplicação dessa estratégia enfrenta obstáculos globalmente, conforme indicado nos estudos internacionais, devido à sua subutilização no processo de diagnóstico e tratamento, e nos fatores motivacionais de natureza pessoal, de gestão e político-econômico. Esses obstáculos refletem em cuidados aquém do desejado para menores de 5 anos, impactando os índices de morbimortalidade por causas evitáveis, que poderiam ser tratadas de forma eficaz na APS^(20,21)^.

A organização da APS em torno das ESFs ampliou acessos e alcançou bons resultados diante da complexidade do processo de saúde-doença da população. Entretanto, a APS no Brasil enfrenta desafios, como a manutenção de características do modelo tradicional, caracterizado pelo pronto atendimento como referência, e fragilidades na coordenação do cuidado, na atenção centrada na família e na longitudinalidade. São muitas as restrições, e a maioria delas remete à desvalorização do Sistema Único de Saúde, à desmotivação nas formações profissionais, à escassez de recursos financeiros, entre outras^(22)^.

Os países que conseguem utilizar a estratégia AIDPI relatam que o tratamento baseado em evidências melhora a qualidade do cuidado, bem como a identificação dos sinais e sintomas para a tomada de decisão do profissional. A AIDPI foi comprovadamente eficaz para melhorar a qualidade da assistência, ao aumentar a economia de custos com saúde e, possivelmente, reduzir a mortalidade infantil em países em desenvolvimento. A atenção estruturada, por meio de gestão integrada abrangente e consistente da infância (AIDPI), tem sido utilizada para promover avaliação e classificação precisas de doenças infantis, além de garantir tratamento combinado adequado, fornecer ao cuidador aconselhamento e resolutividade, especialmente nos encaminhamentos, e contribuir diretamente para redução da mortalidade e morbidade infantil^(23)^.

O meio rural foi um fator protetivo para o desenvolvimento de agravos. Evidencia-se internacionalmente que, em territórios com áreas rurais onde o atendimento é feito por enfermeiros, a AIDIP é utilizada e foi elogiada por identificar sistematicamente sinais de perigo e encaminhar essas crianças quando necessário, mostrando-se eficaz^(20)^. No Brasil e em alguns países, os achados divergem, pois os moradores de áreas rurais têm acesso deficitário a cuidados, e apresentam piores condições de saúde, quando comparados à população urbana. Como dificuldades, evidenciam-se o transporte para locomoção e a comunicação. Primeiro esgotam-se as opções locais, para depois buscar o atendimento em saúde fora da comunidade. Em contrapartida, observa-se que, em algumas comunidades, a população rural tem a disponibilidade do serviço de saúde próximo, mas escolhe viajar até centros maiores para buscar tratamento^(24)^. Esse deslocamento pode estar atrelado também à qualidade do atendimento, especialmente do atendimento médico oferecido à população em cenários rurais. Estudo internacional aponta que, nesses serviços, diagnósticos inadequados, uso irracional de antibióticos e outros medicamentos e polifarmácia foram considerados padrão para a prática médica na APS, o que foge do modelo de promoção e prevenção e conduta ao modelo de cuidado curativo^(25)^.

Evidenciou-se que ter agravo no período neonatal pode ser considerado um fator protetivo à medida que essas crianças podem ser reconhecidas/vistas pela equipe e, assim, reduzir o risco para agravamento ou desenvolvimento de agravos ao longo do primeiro ano de vida, além de destacar o enfermeiro como profissional de atendimento nos primeiros meses. Neste estudo, observa-se que 57,1% dos cuidadores utilizam consistentemente o mesmo serviço de saúde para o atendimento das crianças, e apresentam uma preferência significativa pelo enfermeiro como o profissional que melhor as conhece^(26)^. Os enfermeiros são vistos como profissionais competentes e acessíveis, com capacidade de oferecer orientações adequadas e estabelecer relação de confiança e vínculo com os cuidadores^(8)^.

Para o presente estudo, observou-se que crianças com acompanhamento regular de puericultura apresentaram mais agravos registrados no primeiro ano de vida. Esse acompanhamento é fundamental para a identificação precoce de problemas de saúde e intervenção oportuna, visto que a puericultura fortalece o reconhecimento desses agravos. Na literatura, o cuidado infantil na APS deixa evidente que a ESF é identificada como serviço de referência pela maioria dos cuidadores, mas ainda há um alto índice de outros serviços da rede assistencial vistos como referência. Ao surgirem novos problemas de saúde, há uma tendência a procurar outro serviço para resolução, o que pode indicar questões relacionadas à percepção da qualidade do atendimento especializado no serviço habitual^(26)^. Também é preocupante que a maioria dos prontuários médicos estivesse relacionada a cuidados infantis, o que mostra que os serviços de APS do município não foram procurados devido a queixas de complicações/agravos à saúde^(16)^. Comportamentos assim ressaltam a importância de fortalecer a APS e garantir a integralidade do cuidado para evitar a fragmentação dos serviços^(26)^.

Isso corrobora estudos internacionais, que mostram que muitos pais ignoram os cuidados primários e preferem levar seus filhos em especialistas (nível secundário ou terciário) e utilizar métodos invasivos, como tratamento hospitalocêntrico e medicalizado, sendo, em muitos casos, as Unidades Básicas de Saúde utilizadas para imunização, acompanhamento de pessoas saudáveis e medidas preventivas^(20)^.

As limitações do estudo estão atreladas ao encontro de prontuários incompletos e/ou sem informações nos relatórios extraídos dos prontuários e/ou a inexistência dos mesmos. Outro fator limitante é a identificação apenas de agravos que foram acolhidos, atendidos e registrados na APS, através da ESF, excluindo aqueles invisibilizados pelo serviço da ESF ou que foram acolhidos em outros pontos da RAS, o que impossibilita generalizações para todas as crianças do cenário geográfico do estudo.

## CONCLUSÃO

Os agravos respiratórios foram os mais prevalentes no primeiro ano de vida e classificados como sensíveis à APS. A maioria dos agravos de saúde inicia após o 2º mês de vida e persiste até o 10º mês. Esse padrão indica a importância de uma vigilância contínua e de intervenções preventivas e terapêuticas adequadas durante o primeiro ano de vida para abordar esses problemas de saúde em momentos críticos. Há fatores de proteção a essa população, como residir em áreas rurais do município assim como ter agravo nos primeiros dias de vida, o que possibilita a visibilidade das crianças e realizar acompanhamento da puericultura na ESF.

Intervenções preventivas, como acompanhamento regular na puericultura, podem facilitar a identificação do agravo, tratamento adequado e vigilância contínua de crianças que apresentam agravos no 1^o^ mês de vida, sendo essenciais para melhorar a saúde infantil. Fica evidente que as equipes de saúde, em especial os enfermeiros, necessitam fortalecer formação para melhorar a prática de cuidado com neonatos e lactentes, especialmente sobre as ações da AIDPI, visto que a maioria dos atendimentos foi realizada pelo médico da ESF. A equipe de saúde, em especial os médicos da ESF, precisa reconhecer a importância de, ao finalizar o atendimento de agravo, transferir essa criança para rotinas de puericultura e atendimento sistematizado, reconhecendo possíveis riscos para o crescimento e desenvolvimento infantil.

É necessária uma organização dos serviços públicos de saúde, com o desenvolvimento de estratégias resolutivas, partindo do diagnóstico estabelecido por este estudo, e que ações de prevenção e fortalecimento da APS possam reconhecer precocemente e ser resolutivas, prevenindo complicações, ICSAP e a implementação fraca da AIDPI.

## Data Availability

Os dados estão disponíveis sob consulta/contato com o autor de correspondência.

# Translation: Incidencia de enfermedades prevalentes en lactantes en Atención Primaria de Salud: estudio de cohort

**DOI:** 10.1590/1980-220X-REEUSP-2024-0358es

**Authors:** Luana Bartsch, Veronica Souza Cavalheiro, Keli Verissimo Couto, Débora Cristina Limberger, Aline Cammarano Ribeiro, Darielli Resta Fontana, Leonardo Bigolin Jantsch

**Affiliations:** 1 Universidade Federal de Santa Maria, Palmeira das Missões, RS, Brasil.; 2 Universidade Federal de Santa Maria, Santa Maria, RS, Brasil.

**Keywords:** Cuidado del Niño, Lactante, Atención Primaria de Salud, Atención Integrada a las Enfermedades Prevalentes de la Infancia

## Abstract

**Objetivo:**

Analizar los problemas de salud prevalentes en lactantes en el primer año de vida en un municipio del norte de Rio Grande do Sul.

**Método:**

Se trata de un estudio de cohorte documental retrospectivo, con búsqueda en historias clínicas electrónicas de servicios de Atención Primaria de Salud de nacidos vivos en el año 2022. Se realizó un seguimiento de estos participantes a través del acceso al informe de la historia clínica electrónica desde el 29 de enero de 2022 hasta el 30 de diciembre de 2023, excluyendo el período neonatal. Los datos se analizaron mediante comparación de frecuencias.

**Resultados:**

Más de la mitad de los niños monitoreados en la cohorte recibieron al menos un tratamiento para una afección infantil prevalente; aproximadamente el 43A tuvo dos citas; y el 30A tuvo tres citas en Atención Primaria en el primer año de vida. La mayoría de los problemas están asociados a afecciones respiratorias, comienzan después del segundo mes de vida y persisten hasta el décimo mes.

**Conclusión:**

Se destaca la importancia del seguimiento continuo y de las intervenciones preventivas y terapéuticas adecuadas durante el primer año de vida y en los momentos críticos.

## INTRODUCCIÓN

La Atención Primaria de Salud (APS), considerada inicialmente como la puerta de entrada al sistema de salud, ha pasado a ocupar un lugar central en la Red de Atención a la Salud (RAS), actuando como punto central de comunicación, organización y coordinación de la atención en salud en Brasil. En este contexto, la estrategia prioritaria para ampliar, fortalecer y cualificar la APS es la Estrategia de Salud de la Familia (ESF). Esta ha alcanzado resultados significativos en términos de indicadores de salud, eficiencia y equidad^(1)^. Sin embargo, es necesario conocer el nivel de resolutividad de la APS, ya que influye en el proceso salud-enfermedad de los individuos, permitiendo identificar fallas en la atención que pueden resultar en complicaciones de salud y hospitalizaciones cuando no son resueltas y atendidas^(2)^.

En el contexto de la salud infantil dentro de la APS, se reconoce que los principales problemas de salud están relacionados con la evaluación del desarrollo y crecimiento infantil, así como la atención a las enfermedades prevalentes de la infancia^(3)^, situaciones que también se reconocen dentro de las rutinas o demandas espontáneas de cuidado infantil.

El cuidado de los niños en la primera infancia es importante, ya que en esta etapa se desarrolla el sistema nervioso y se adquieren habilidades que facilitan el desarrollo de capacidades futuras. Además, destaca la importancia del periodo neonatal (de cero a 28 días) y los primeros mil días, una fase de desarrollo neurológico, emocional, cognitivo y psicomotor único. Sin embargo, es en este grupo de edad que los niños pueden ser susceptibles a enfermedades y problemas de salud^(4)^.

La salud infantil ha sido una prioridad en las agendas políticas de Brasil durante décadas, lo que ha dado lugar a avances significativos. Un hito importante en este contexto fue la introducción, en 1996, de la estrategia de Atención Integral a las Enfermedades Prevalentes de la Infancia (AIEPI), que se utiliza para supervisar la atención de la salud infantil en la APS. Esta estrategia pretende promover la promoción de la salud, la prevención de las enfermedades, la detección precoz y el tratamiento eficaz de los principales problemas de salud que afectan a los niños. La implantación de la AIEPI se ha asociado a una reducción significativa de las tasas de mortalidad infantil por estas enfermedades, y ha contribuido a garantizar el pleno desarrollo infantil, apoyando la investigación para identificar y tratar las enfermedades infantiles que confluyen mayoritariamente con las Internaciones por Condiciones Sensibles a la Atención Primaria (ICSAP)^(3)^.

En 2015, se logró un avance significativo en la salud infantil con la publicación de la Ordenanza n.º 1.130, que estableció la Política Nacional de Atención Integral a la Salud Infantil (PNAISC en la sigla en portugués). La PNAISC está estructurada en ejes. Destaca el eje IV «Atención integral a niños con enfermedades prevalentes de la infancia y enfermedades crónicas», que aborda las enfermedades de la infancia con una perspectiva más amplia, así como las enfermedades crónicas, con el objetivo de desarrollar estrategias, diagnosticar precozmente y proporcionar una atención cualificada^(5,6)^.

Según la estrategia AIEPI, las enfermedades prevalentes son un conjunto de enfermedades con mayor incidencia en la infancia. Las más comunes son: enfermedades respiratorias, como tos o dificultad para respirar, que se asocian a neumonía, sibilancias, infección de oído aguda o crónica e infección de garganta; enfermedades febriles, incluidas las enfermedades febriles y la malaria en zonas de riesgo; enfermedades diarreicas, como deshidratación, diarrea persistente y disentería; y enfermedades nutricionales, relacionadas con malnutrición, anemia y otros problemas de crecimiento^(7)^.

Entre esas afecciones, la literatura indica que las enfermedades respiratorias están entre las principales causas de morbilidad infantil en Brasil, y se destacan como causas de internación hospitalaria por afecciones sensibles a la APS. Esto resalta la importancia de un sistema de APS robusto y eficaz para la prevención y el manejo de estas enfermedades, reduciendo así la necesidad de hospitalizaciones^(8)^. Cabe destacar que la literatura incluye la estrategia AIEPI como práctica de enfermería durante el cuidado de los niños y que aún existe una prevalencia de muertes infantiles por causas evitables que podrían prevenirse y tratarse con acciones sanitarias y comportamientos calificados basados en AIEPI^(9)^. Sin embargo, para ello, es necesario identificar las principales enfermedades que se tratan, su incidencia y los factores de riesgo para la población menor de 1 año, el grupo de edad con mayor vulnerabilidad en la infancia^(10)^. Para ello, este estudio tiene como objetivo analizar los problemas de salud prevalentes en lactantes en su primer año de vida en un municipio del norte de Rio Grande do Sul.

## MÉTODO

### Tipo de Estudio

Se trata de un estudio originado en un proyecto de investigación matriz titulado «Problemas de salud y seguimiento de lactantes en la primera infancia: un estudio de cohortes», que es un estudio de cohortes ambidiestro (retrospectivo y prospectivo) y documental.

### Localización, Población y Criterios de Selección

El sitio del estudio es el municipio de Palmeira das Missões, localizado en la región noroeste de Rio Grande do Sul, con una población de 33.216^(11)^. La red municipal de atención a la salud está compuesta por 11 ESF, lo que representa una cobertura del 100%.

Se trata de un estudio de base poblacional que incluyó como participantes la cohorte de nacidos vivos en el municipio de Palmeira das Missões, RS, que nacieron en 2022 (01 de enero a 31 de diciembre de 2022) y vivían en el municipio en el momento de la recogida de datos. Según el informe proporcionado por el Departamento Municipal de Salud, hubo 383 nacimientos de residentes en Palmeira das Missões en 2022 (N = 383).

Los participantes fueron reclutados accediendo a la lista de nacidos vivos (que contiene el nombre completo del niño, el nombre de la madre y la fecha de nacimiento). Se excluyeron aquellos que, en el momento de la recogida de datos, tenían domicilio fuera del municipio, escenario del estudio, totalizando 350 participantes (N = 350) al final de la recogida de datos.

### Recolección de Datos

Los datos se recogieron accediendo a las historias clínicas electrónicas de los servicios de salud del municipio, E-SUS (historias clínicas de APS). Para el propósito de este estudio, los datos sobre el seguimiento y las enfermedades infantiles se recogieron en cada acceso/registro en las historias clínicas electrónicas de los participantes, desde el 29 de enero de 2022 hasta el 30 de diciembre de 2023, dado que este era el período en el que los niños tenían entre 28 y 365 días de edad, con el fin de excluir el período neonatal. La herramienta utilizada para la recolección de datos fue la plataforma *Google Forms,* que genera electrónicamente una hoja de cálculo *Excel*.

La herramienta de recolección de datos fue un formulario semiestructurado basado en los manuales del Ministerio de Salud^(12)^, el manual AIEPI (versión 2012 (neonatal) y versión 2017 (2 meses a 5 años)) y literatura relevante en el área^(13)^. Las variables utilizadas fueron: la caracterización demográfica (urbana/rural) y materna (edad materna); el sexo del niño (masculino y femenino) y el color/raza (blanco/no blanco); el número y la frecuencia recomendada de las citas de seguimiento en la guardería; el profesional que realizó la atención; y las enfermedades en la primera infancia, con el resultado analizado en este estudio (desarrollo de una enfermedad prevalente en el primer año de vida), clasificadas según la Clasificación Internacional de Atención Primaria (CIAP) o la Clasificación Internacional de Enfermedades (CIE) proporcionada por el profesional que realizó la atención. Esta CIE o CIAP fue clasificada según las condiciones descritas en el manual de AIEPI por los investigadores del estudio, bajo la supervisión del investigador responsable. Al tratarse de un estudio documental que analizaba los registros de atención en APS, no se tuvieron en cuenta las pérdidas de seguimiento.

### Análisis de los Datos

Los datos se analizaron desde una perspectiva de caracterización en formato descriptivo (frecuencia relativa (%) y frecuencia absoluta (n)). Para comparar las variables independientes, como la edad materna, el lugar de residencia, el color/raza y la frecuencia de las citas de seguimiento en la guardería, con los resultados analizados en este estudio (desarrollo de una afección prevalente en el primer año de vida), se utilizaron la comparación de frecuencias y el riesgo relativo, en función de la característica de la variable, así como la comparación de medias y la prueba T, definidas tras comprobar la normalidad de los datos. Se utilizó la prueba de Kruskal-Wallis para comparar la edad de la primera lesión con el tipo de lesión, la conducta y el profesional cuidador.

### Aspectos Éticos

El proyecto fue aprobado por el Comité de Ética en Investigación de la Universidad Federal de Santa María, *Campus* Palmeira das Missões, bajo el Dictamen nº 6.494.336 y Certificado de Sometimiento a Evaluación Ética 74518823.9.00005346, en octubre de 2023. La investigación cumple con la Resolución 466 del 12 de diciembre de 2012 y la Ley General de Protección de Datos Personales, Ley 13.709/2018, especialmente el artículo 7 («IV - para los estudios realizados por organismos de investigación, garantizando, siempre que sea posible, la anonimización de los datos personales»). Como instrumento de consentimiento se utilizó el Formulario de Consentimiento Informado.

## RESULTADOS

De las 350 historias clínicas analizadas, 52% eran de niñas, y la mayoría eran de raza/color blanca (96%), residentes en el área urbana del municipio (94%). La edad materna media fue de 28 años (DE ± 6,5 años). En cuanto a la caracterización de los cuidados prestados entre los 28 y los 364 días de vida en esta cohorte de seguimiento, la Tabla 1 describe el número de visitas y la edad media en las visitas.

**Tabla 1:** Caracterización del número de consultas en el primer año de vida y clasificación de las enfermedades según la atención integral a las enfermedades prevalentes de la infancia – Palmeira das Missões, RS, Brasil, 2024.

Número de asistencias	n(%)	Media (meses)	Mínimo-máximo (meses)
Una asistencia	192(54,9)	4,9(3,1)	1–11,7
Dos asistencias	150(42,9)	5,8(2,9)	1,1–11,9
Tres asistencias	15(30,0)	7,0(2,9)	1,4–11,76
Cuatro asistencias	66(18,9)	7,5(3,1)	1,11
Cinco asistencias	45(12,9)	8,3(2,6)	0,9–11,37
Seis asistencias	20(5,7)	8,6(2,9)	2,9–11,9
Siete asistencias	11(3,1)	8,3(3,0)	3,3–11,6
Ocho asistencias	8(2,3)	8,1(2,9)	3,7–11,7
Nueve asistencias	5(1,4	8,7(1,7)	6,1–10,2
Diez o + asistencias	17(4,8)	9,9(1,5)	8,3–11,9
**Enfermedades (Atención Integral a las Enfermedades Prevalentes de la Infancia)**			
Disfunción respiratoria	283(52,4)		
Diarrea	30(5,6)		
Fiebre	45(8,3)		
Problema en el oído	12(2,2)		
Dolor de garganta	11(2,0)		
Desnutrición/anemia/problema de crecimiento	14(2,6)		
Alergias	35(6,5)		
Otros	110(20,4)		

Más de la mitad de los niños seguidos en la cohorte recibieron al menos una visita por una afección prevalente en la infancia; alrededor del 43% recibió dos visitas; y el 30%, tres visitas en el primer año de vida. La mayoría de los problemas estaban relacionados con disfunciones respiratorias. La Tabla 2 muestra la relación entre el tipo de afección y el profesional que realizó la atención.

**Tabla 2:** Relación entre la patología en la atención integral a las enfermedades prevalentes de la infancia y el profesional que realizó la atención en atención primaria – Palmeira das Missões, RS, Brasil, 2024.

Problema de salud	Enfermería	Médico de ESF	Pediatra	Total	Valor de p*
Disfunción respiratoria	54(19,1)	187 (66,1)	42(14,8)	283(100,0)	<0,001
Diarrea	5(16,7)	21(70,0)	4(13,3)	30(100,0)	
Fiebre	9(20,0)	28(62,2)	8(17,8)	45(100,0)	
Problema en el oído	1(8,3)	7(58,3)	4(33,3)	12(100,0)	
Dolor de garganta	5(45,5)	6(54,5)	0(0,0)	11(100,0)	
Desnutrición/anemia/problema de crecimiento	3(21,4)	7(50,0)	4(28,6)	14(100,0)	
Alergias	12(34,3)	16(45,7)	7(20,0)	35(100,0)	
Otros	45(40,09)	49(44,5)	16(14,5)	110(100,0)	
**Total**	**134(24,8)**	**321(59,4)**	**85(15,7)**	**540(100,0)**	

Claves: ESF – Estrategia Salud de la Familia

*Test Chi-quadrado.

En el análisis entre el problema AIEPI y el profesional que realizó la atención, destaca que el profesional médico de la ESF trató más problemas relacionados con disfunciones respiratorias (66,1), diarreas (70,0), fiebre (62,2) y problemas de oído (58,3). Las enfermeras atendieron más los dolores de garganta (45,5) y las alergias (34,3). Por su parte, el pediatra atendió problemas de oído (33,3) y desnutrición/anemia/problemas de crecimiento (28,6). En conjunto, el médico de la ESF fue el que más atención prestó, con casi el 60%, seguido de la enfermera, con aproximadamente el 25%, y el pediatra, con el 15,7%. La Tabla 3 describe los factores de riesgo identificados para el desarrollo de al menos un problema de salud en el primer año de asistencia en APS.

**Tabla 3:** Factores de riesgo para el desarrollo de problemas de salud en el primer año de vida – Palmeira das Missões, RS, Brasil, 2024.

	Problema de salud 1º ano		RR(IC)	Valor de p***
	Si	No		
Problema neonatal*				<0,001
*Si*	3(9,4)	29(90,6)	1	
*No*	189(59,4)	129(40,6)	14,163 (4,225–47,474)	
Sexo				0,933
*Masculino*	92(55,1)	75(44,9)	1,018(0,668–1,552)	
*Femenino*	100(54,6)	83(45,4)	1	
Local de residencia				
*Urbano*	185(56,4)	143(43,6)	2,772(1,101–6,976)	0,025
*Rural*	7(31,8)	15(68,2)	1	
Raza/color				0,706
*Blanca*	183(55,1)	149(44,9)	1	
*No blanca*	7(50,0)	7(50,0)	0,814(0,279–2,373)	
Puericultura en el 1^er^ mes*	53(39,6)	81(60,4)	1,446(0,934–2,238)	0,061
Puericultura en el 2º mes*	75(65,2)	40(34,8)	1,891(1,192–2,999)	0,006
Puericultura en el 4º mes*	53(66,3)	27(33,8)	1,850(1,098–3,116)	0,020
Puericultura en el 6º mes*	55(65,5)	29(34,5)	1,7886(1,072–2,974)	0,025
Puericultura en el 9º mes*	40(72,7)	15(27,3)	2,509(1,328–4,738)	0,004
Puericultura en el 12º mes*	63(71,6)	25(28,4)	2,598(1,540–4,382)	<0,001

Claves *Haber desarrollado una condición atendida en la APS en el período neonatal

**Haber realizado un número adecuado de consultas según el Ministerio de Salud, Brasil

***Prueba de Chi cuadrado; IC: Intervalo de Confianza; RR: Riesgo Relativo.

Los niños que no desarrollaron una enfermedad en el periodo neonatal tienen un riesgo 14 veces mayor de desarrollar una enfermedad durante el primer año de vida. También existe una diferencia significativa en cuanto al lugar de residencia, con un riesgo aproximadamente 3 veces mayor para los que viven en zonas urbanas en comparación con los que viven en zonas rurales. Por lo que se refiere al seguimiento de las guarderías, es evidente que los niños con un mayor número de citas en la guardería también presentaban un mayor riesgo de padecer problemas de salud. La Tabla 4 muestra los procedimientos adoptados para cada problema de salud en el primer año de vida y el profesional que orientó el procedimiento.

**Tabla 4:** Conductas adoptadas para los problemas de salud en el primer año de vida según el profesional que las orientó – Palmeira das Missões, RS, Brasil, 2024.

	Conducta del profesional	
Problema de salud	Alta del episodio (sin referencia)	Referido para puericultura o servicio especializado
Disfunción respiratoria	167(65,2)	89(34,8)
Diarrea	21(70,0)	9(30,0)
Fiebre	34(77,3)	10(22,7)
Problema en el oído	7(58,3)	5 (41,7)
Dolor de garganta	8(72,7)	3(27,3)
Desnutrición/anemia/problema de crecimiento	6(50,0)	6(50,0)
Alergias	17(58,6)	12(41,4)
Otros	46(44,2)	58(55,8)
**Profesional que realizó la atención**
Médico de la Estrategia de Salud de la Familia	259(78,5)	71(21,5)
Enfermero	54(39,7)	82(60,3)
Pediatra	31(34,8)	58(65,2)

El comportamiento después de la asistencia varió en función de la clasificación AIEPI del problema. La gran mayoría de los pacientes fueron dados de alta (sin derivación) por disfunción respiratoria (65,2), diarrea (70,0), fiebre (77,3), problemas de oído (58,3) y dolores de garganta (72,7). La malnutrición/anemia/problemas de crecimiento supuso el 50% de las altas y derivaciones para seguimiento en guardería, siendo «otros» (55,8) la más frecuentemente derivada para seguimiento en guardería o servicios especializados. La Figura 1 muestra la edad de consulta relacionada con la afección en el primer año de vida y el profesional que realizó la atención.

**Figura 1:** Relación entre edad en la consulta, problema de salud y profesional que prestó atención en la primera consulta. Palmeira das Missões, Rio Grande do Sul, Brasil, 2024.

Claves: 1 – respiratorio; 2 – diarrea; 3 – fiebre; 4 – problema de oído; 5 – dolor de garganta; 6 – desnutrición/anemia/problema de crecimiento; 7 – alergias; 8 – otros.

Los problemas de salud en los niños muestran diferentes patrones de prevalencia a lo largo de los primeros meses de vida. Por término medio, las disfunciones respiratorias se concentran en el 5º mes de vida. La malnutrición/anemia/problemas de crecimiento comienzan en el 1^er^ mes y duran hasta el 10º mes, con un pico de prevalencia en el 6º mes. La diarrea, la fiebre y los problemas de oído son más frecuentes del 6º al 8º mes de vida, y los dolores de garganta tienen una mayor prevalencia en el 4º mes. Las edades en la primera consulta varían significativamente (p < 0,001) en función del profesional. Las consultas con pediatras tienden a producirse más tarde (de media) en comparación con las consultas con enfermeras y médicos de ESF.

## DISCUSIÓN

Más de la mitad de los lactantes tuvieron al menos una consulta con la ESF por un problema de salud en el primer año de vida, la mayoría asociados a problemas respiratorios, con predominio de las consultas con el médico del SFS. Factores como tener un problema en el primer mes de vida y vivir en zonas rurales se consideraron protectores. El seguimiento en la guardería es un factor determinante a la hora de pedir cita por problemas infantiles en el primer año de vida. Las enfermeras son los profesionales que atienden a los lactantes más pequeños, en comparación con las demandas de los médicos y pediatras de la ESF. En cuanto a las derivaciones, los resultados están en línea con el bajo nivel de adherencia a la atención infantil por parte de los médicos de la ESF, reflejo de la calidad de la formación de pregrado, del modelo actual, que puede no incluir todas las competencias esenciales para una adecuada atención infantil en APS, así como de la ausencia o ineficacia de programas de formación continuada durante el ejercicio profesional. Además, la práctica de los médicos de familia de derivar a los niños a la medicina preventiva y a la puericultura es aún incipiente^(14)^.

La infancia es un periodo en el que pueden surgir muchos problemas de salud. Más de la mitad de los lactantes tuvieron al menos un problema de salud en el primer año de vida, y la mayoría se asociaron a afecciones respiratorias, según los resultados de este estudio. En la literatura, las infecciones respiratorias agudas son una de las principales causas de morbilidad y mortalidad infantil, y la región sur presenta las mayores tasas de enfermedades respiratorias debido al clima estacional^(15)^, corroborando el estudio que destaca las enfermedades respiratorias como las principales causas de morbilidad infantil en Brasil^(8)^.

Los niños menores de 1 año presentan el mayor número de hospitalizaciones por enfermedades respiratorias, debido a la inmadurez del sistema inmunológico, a las características del propio aparato respiratorio en los primeros 12 meses de vida, a la interrupción de la lactancia materna y al inicio de las actividades de guardería^(13,14)^. Según este estudio, aproximadamente el 81,5% de los lactantes tuvieron algún problema de salud en el primer año de vida, y el 93,3% en el segundo año de vida, siendo los problemas más frecuentes los relacionados con el aparato digestivo y respiratorio. Además, el 24,7% de los lactantes de 2 años ya habían sido hospitalizados al menos una vez, y los niños que no recibieron lactancia materna exclusiva hasta los 6 meses o que no recibieron leche materna hasta el primer año de vida presentaron una mayor prevalencia de problemas de salud prevalentes^(16)^.

Mientras tanto, la literatura internacional en el Reino Unido destaca que la mayoría de las atenciones a niños menores de 5 años en APS son por afecciones virales agudas y autolimitadas, es decir, que se resuelven sin intervención médica y podrían ser manejadas por profesionales sanitarios, y el motivo más común de hospitalizaciones también se debió a infecciones del tracto respiratorio superior (14%) y tos (7%)^(17)^.

Se calcula que en todo el mundo se producen 33 millones de episodios de infección aguda de las vías respiratorias inferiores asociada al virus respiratorio sincitial (VRS) en niños de 0 a 60 meses. Los lactantes se ven afectados en todas las regiones, pero la incidencia aguda de la infección de las vías respiratorias inferiores asociada al VRS alcanza su máximo en los países de ingresos bajos y medios bajos en los niños menores de 3 meses y en los países de ingresos medios altos y altos entre los 3 y los 6 meses. Se observa una media de cinco meses, que es el periodo con mayor incidencia de problemas respiratorios, lo que corrobora el hecho de que muchos niños empiezan en guarderías/escuelas debido a que sus madres se reincorporan al trabajo^(18)^. En un estudio realizado en España con 51.292 niños menores de 5 años también se observó que en el primer año de vida se producía el mayor número de infecciones respiratorias agudas y por VRS (134,4 por 1.000 niños)^(19)^.

Por otro lado, es importante destacar la eficacia de la APS, ya que tiene un gran impacto en la prevención e identificación precoz de estas enfermedades, especialmente las respiratorias en la infancia^(15)^. Cabe destacar que las enfermedades prevalentes de la infancia pueden ser identificadas precozmente a través de la puericultura, que debe identificar los signos precoces de las principales enfermedades y recomendar las acciones más eficaces, formando un vínculo con la familia y teniendo la oportunidad, como enfermera, de apoyar y orientar a la familia para reconocer precozmente las enfermedades^(16)^.

Mientras tanto, la literatura señala los desafíos en la implementación de la AIEPI, que a pesar de haber sido introducida hace mucho tiempo, aún no es ampliamente adoptada en el manejo de las enfermedades prevalentes de la infancia en el ámbito de la APS. La aplicación de esta estrategia se enfrenta globalmente a obstáculos, como indican estudios internacionales, debido a su infrautilización en el proceso de diagnóstico y tratamiento, y a factores motivacionales de carácter personal, de gestión y político-económicos. Estos obstáculos se traducen en una atención inferior a la deseable para los niños menores de 5 años, lo que repercute en las tasas de morbilidad y mortalidad debidas a causas prevenibles que podrían tratarse eficazmente en la APS^(20,21)^.

La organización de la APS en torno a la ESF ha ampliado el acceso y obtenido buenos resultados frente a la complejidad del proceso salud-enfermedad de la población. Sin embargo, la APS en Brasil enfrenta desafíos, como el mantenimiento de rasgos del modelo tradicional, caracterizado por la atención de emergencia como referencia, y debilidades en la coordinación de la atención, la atención centrada en la familia y la longitudinalidad. Hay muchas restricciones, la mayoría de las cuales se refieren a la desvalorización del Sistema Único de Salud, la desmotivación en la formación profesional y la falta de recursos financieros, entre otras^(22)^.

Los países que logran utilizar la estrategia AIEPI informan que el tratamiento basado en la evidencia mejora la calidad de la atención, así como la identificación de signos y síntomas para la toma de decisiones profesionales. La AIEPI ha demostrado su eficacia para mejorar la calidad de la atención, aumentar el ahorro de costes sanitarios y posiblemente reducir la mortalidad infantil en los países en desarrollo. La atención estructurada, mediante una gestión integrada de la infancia completa y coherente, ha servido para promover una evaluación y clasificación precisas de las enfermedades infantiles, así como para garantizar un tratamiento combinado adecuado, proporcionar a los cuidadores asesoramiento y capacidad resolutiva, especialmente en las derivaciones, y contribuir directamente a reducir la mortalidad y morbilidad infantiles^(23)^.

El medio rural fue un factor protector para el desarrollo de enfermedades. Existen pruebas internacionales de que, en las zonas rurales donde los cuidados son prestados por enfermeras, se utiliza el AIDIP, que ha sido elogiado por identificar sistemáticamente los signos de peligro y derivar a esos niños cuando es necesario, demostrando su eficacia^(20)^. En Brasil y en algunos otros países, los resultados difieren, ya que los residentes de las zonas rurales tienen un acceso deficiente a la atención y peores condiciones de salud en comparación con la población urbana. Las dificultades incluyen el transporte y la comunicación.

Primero se agotan las opciones locales y luego se busca atención en salud fuera de la comunidad. Por otra parte, se ha observado que, en algunas comunidades, la población rural dispone de un servicio sanitario cercano, pero opta por desplazarse a centros más grandes para buscar tratamiento^(24)^. Este desplazamiento también puede estar relacionado con la calidad de la asistencia, especialmente de la atención médica ofrecida a la población en entornos rurales. Un estudio internacional señala que, en estos servicios, los diagnósticos inadecuados, el uso irracional de antibióticos y otros medicamentos y la polifarmacia se consideraban prácticas médicas habituales en la APS, lo que se aparta del modelo de promoción y prevención y conduce al modelo de atención curativa^(25)^.

Se ha demostrado que tener un problema en el periodo neonatal puede considerarse un factor protector, ya que estos niños pueden ser reconocidos/vistos por el equipo y reducir así el riesgo de empeoramiento o desarrollo de problemas durante el primer año de vida, además de destacar a las enfermeras como profesionales de los cuidados en los primeros meses. En este estudio se constató que el 57,1% de los cuidadores recurren sistemáticamente al mismo servicio sanitario para atender a sus hijos, y tienen una preferencia significativa por las enfermeras como los profesionales que mejor los conocen^(26)^. Las enfermeras se consideran profesionales competentes y accesibles, con capacidad para ofrecer una orientación adecuada y establecer una relación de confianza y vinculación con los cuidadores^(8)^.

En este estudio, se observó que los niños con un seguimiento regular presentaban más problemas en el primer año de vida. Este seguimiento es fundamental para la identificación precoz de problemas de salud y la intervención oportuna, ya que el cuidado infantil refuerza el reconocimiento de estos problemas. La literatura sobre atención infantil en APS muestra que la ESF es identificado como servicio de referencia por la mayoría de los cuidadores, pero sigue habiendo un alto índice de otros servicios de la red asistencial vistos como referencia. Cuando surgen nuevos problemas de salud, hay una tendencia a buscar otro servicio para su resolución, lo que puede indicar cuestiones relacionadas con la percepción de la calidad de la atención especializada en el servicio habitual^(26)^. También es preocupante que la mayoría de las historias clínicas estuvieran relacionadas con el cuidado de los niños, lo que muestra que no se buscaban los servicios de APS del municipio por quejas de complicaciones/enfermedades de salud^(16)^. Comportamientos como este resaltan la importancia de fortalecer la APS y garantizar la atención integral para evitar la fragmentación de los servicios^(26)^.

Esto corrobora estudios internacionales, que demuestran que muchos padres ignoran la atención primaria y prefieren llevar a sus hijos a especialistas (de nivel secundario o terciario) y utilizar métodos invasivos, como el tratamiento centrado en el hospital y medicalizado, mientras que en muchos casos las Unidades de Atención Primaria se utilizan para la inmunización, el seguimiento de personas sanas y las medidas preventivas^(20)^.

Las limitaciones del estudio están relacionadas con el hallazgo de historias clínicas incompletas y/o sin información en los informes extraídos de las historias clínicas y/o su inexistencia. Otro factor limitante es la identificación solamente de enfermedades que fueron recibidas, tratadas y registradas en APS, a través de la ESF, excluyendo aquellas que fueron invisibilizadas por el servicio de la ESF o que fueron recibidas en otros puntos de la RAS, lo que imposibilita la generalización sobre todos los niños del ámbito geográfico del estudio.

## CONCLUSIÓN

Los problemas respiratorios fueron los más prevalentes en el primer año de vida y se clasificaron como sensibles a la APS. La mayoría de los problemas de salud comienzan después del 2º mes de vida y persisten hasta el 10º mes. Este patrón indica la importancia de la vigilancia continua y de las intervenciones preventivas y terapéuticas adecuadas durante el primer año de vida para abordar estos problemas de salud en los momentos críticos. Existen factores protectores para esta población, como el hecho de vivir en zonas rurales del municipio, así como el hecho de padecer una enfermedad en los primeros días de vida, lo que hace posible ver a los niños y llevar a cabo la atención infantil en la ESF.

Las intervenciones preventivas, como el cuidado regular de los niños, pueden facilitar la identificación del problema, el tratamiento adecuado y la vigilancia continua de los niños que presentan problemas en el primer mes de vida, y son esenciales para mejorar la salud de los niños. Es evidente que los equipos sanitarios, especialmente las enfermeras, necesitan recibir formación para mejorar su práctica de atención a los neonatos y lactantes, especialmente en lo que respecta a las acciones de AIEPI, dado que la mayor parte de la atención fue prestada por el médico de la ESF. Es necesario que el equipo de salud, especialmente los médicos de la ESF, reconozcan la importancia de transferir al niño a la atención rutinaria y sistemática, reconociendo los posibles riesgos para el crecimiento y el desarrollo del niño.

Es necesario organizar los servicios de salud pública, con el desarrollo de estrategias resolutivas, a partir del diagnóstico establecido por este estudio, para que las acciones preventivas y el fortalecimiento de la APS puedan ser reconocidos precozmente y ser resolutivos, evitando complicaciones, las ICSAP y la débil implementación de la AIEPI.

## Data Availability

Los datos están disponibles a solicitud o contato con el autor correspondiente.
